# Cardiomyocyte Remodeling in Atrial Fibrillation and Hibernating Myocardium: Shared Pathophysiologic Traits Identify Novel Treatment Strategies?

**DOI:** 10.1155/2015/587361

**Published:** 2015-06-29

**Authors:** Brian R. Weil, Cevher Ozcan

**Affiliations:** ^1^Division of Cardiovascular Medicine, University at Buffalo School of Medicine and Biomedical Sciences, Buffalo, NY, USA; ^2^Section of Cardiology, The University of Chicago Medicine, 5841 S. Maryland Avenue, MC6080, Chicago, IL 60637, USA

## Abstract

Atrial fibrillation (AF) is the most common arrhythmia and is associated with a high risk of morbidity and mortality. However, there are limited treatment strategies for prevention of disease onset and progression. Development of novel therapies for primary and secondary prevention of AF is critical and requires improved understanding of the cellular and molecular mechanisms underlying the AF disease process. Translational and clinical studies conducted over the past twenty years have revealed that atrial remodeling in AF shares several important pathophysiologic traits with the remodeling processes exhibited by hibernating myocardium that develop in response to chronic ischemia. These shared features, which include an array of structural, metabolic, and electrophysiologic changes, appear to represent a conserved adaptive myocyte response to chronic stress that involves dedifferentiation towards a fetal phenotype to promote survival. In this review, we discuss the pathophysiology of AF, summarize studies supporting a common remodeling program in AF and hibernating myocardium, and propose future therapeutic implications of this emerging paradigm. Ultimately, better understanding of the molecular mechanisms of atrial myocyte remodeling during the onset of AF and the transition from paroxysmal to persistent stages of the disease may facilitate discovery of new therapeutic targets.

## 1. Introduction

**Figure 1:**
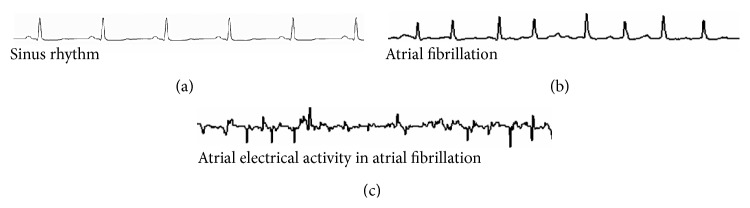
Surface electrocardiogram shows sinus rhythm (a) with organized atrial electrical activity and contraction following impulse formation from the sinus node. There is a one-to-one relationship between atrial (p wave) and ventricular depolarization (QRS) with normal electrical conduction. However, atrial fibrillation (b) is associated with rapid, chaotic atrial electrical activity with variable ventricular conduction. Intracardiac recording of atria (c) shows disorganized electrical activity.

Atrial fibrillation (AF) is the most common sustained arrhythmia, affecting about 1% of the general US population [1]. It is an abnormal heart rhythm characterized by rapid, irregular, and heterogeneous atrial electrical activity that is associated with ineffective atrial contraction (Figure 1) [1]. There is variable and irregular conduction of atrial electrical activity to the distal electrical system and ventricles.

The incidence of AF increases with advancing age, such that 8% of adults older than 80 years are affected by the disease. It is projected that the prevalence of AF will reach 5.6 to 12.1 million individuals in 2050 [2, 3]. Lifetime risks for development of AF are 1 in 4 for men and women 40 years of age and older [4]. Also, AF is associated with an increased risk of all-cause mortality and morbidity including stroke, heart failure, dementia, embolic events, and impaired quality of life. As a result, it is the most common cause of hospital admission for cardiac rhythm disturbances and a major public health problem, with a total annual economic burden of ~$7.9 billion [5–7].

**Figure 2:**
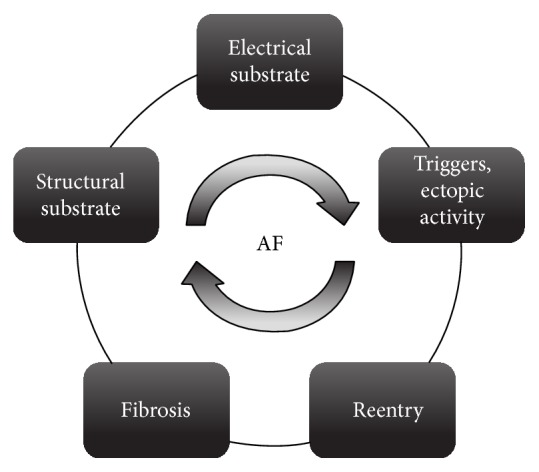
The pathophysiology of atrial fibrillation is complex and includes multiple components. Electrical and structural substrates have a significant role in initiation and progression of atrial fibrillation while closely interacting with several other factors.

Despite the magnitude of the disease, the precise molecular mechanisms underlying AF remain incompletely understood. Elucidating disease mechanisms at the basic and clinical level is essential to identify novel targets for prevention and treatment of AF. To date, studies have revealed that the pathophysiology of AF is complex and includes multiple components ranging from vulnerable atrial substrate to electrophysiological triggers (Figure 2) [1]. Electroanatomical remodeling of atrial myocytes is an essential component of AF pathogenesis and exhibits features that are similar to those of another cardiac pathology, hibernating myocardium (HM). HM is characterized by an array of structural, metabolic, and electrophysiologic changes that have been suggested to represent myocyte dedifferentiation and a conserved adaptive response to stress, which may also be apparent in atrial myocytes during AF. The adoption of a phenotype characteristic of HM by atrial myocytes may contribute to the initiation and progression of AF. Thus, further understanding of the common mechanisms underlying AF and HM may facilitate the development of novel targets for disease prevention and treatment.

The goal of this review, therefore, is to demonstrate the shared pathophysiologic traits of AF and viable dysfunctional myocardium, with particular emphasis on atrial cardiomyocyte remodeling in AF and the characteristics that are shared with myocardial hibernation. Future opportunities to investigate the molecular mechanisms and develop novel therapies for AF are also discussed. To identify relevant articles, a literature search of PubMed-indexed articles ranging from 1948 through October 1, 2014, was performed using the key words “atrial fibrillation” and “hibernation,” “mechanisms,” or “pathophysiology” published in English. All references in this search list were reviewed by the titles and abstracts to select relevant articles for full-text review. Retrieved relevant articles are included in this paper. Thus, the list of references reflects an overview of existing literature relevant to AF and hibernation in atrial myocardium.

## 2. Pathogenesis of Atrial Fibrillation

The pathophysiology of AF is complex and often multifactorial, generally involving an electroanatomical substrate, abnormal impulse formation and/or propagation, focal and dynamic triggers, reentry, and fibrosis in atrial myocardium (Figure 2). Various pathophysiologic mechanisms and multiple disease pathways have been studied in AF; however common molecular mechanisms underlying the initial development of AF and transition from paroxysmal to persistent AF remain unclear. While recent studies have identified a familial form of AF [8], the majority of patients with AF have the common form with no defined genetic susceptibility. Here, we summarize recent findings in AF pathogenesis, particularly those related to the structural and electrical remodeling that occurs throughout the disease process.

### 2.1. Atrial Morphological Changes form a Structural Substrate for AF

Several characteristic atrial architectural changes are typically observed in preclinical models of AF and in patients suffering from the disease. These include inflammation, cell hypertrophy, atrial dilation, and fibrosis, which cumulatively contribute to abnormal electrical signal formation and conduction as an arrhythmogenic substrate [9]. These changes are commonly the result of other underlying heart diseases, such as coronary artery disease, hypertension, valvular disease, and cardiomyopathies, which exert adverse effects on myocyte structure and/or function, predominately via elevations in atrial pressure and wall stress [1]. The elevated hemodynamic load on the atria promotes cellular hypertrophy, cardiomyocyte dysfunction, and disorganization of gap junctions. This process is associated with myocyte death through apoptosis and necrosis. Myocyte loss, together with neurohumoral signaling activated by atrial stretch, prompts extensive replacement fibrosis in large part because of the greater number of fibroblasts present in the atria and its subsequent propensity for fibrotic tissue deposition [1].

Collectively, the aforementioned morphological changes contribute to the formation of a structural substrate that promotes the onset and progression of AF. For instance, loss of atrial cardiomyocyte mass with apoptosis or necrosis causes accumulation of fibrotic tissue, disruption of cell-to-cell communication, diminished conduction velocity, and heterogeneous conduction patterns [9–12]. This replacement fibrosis, along with amyloidosis, inflammation, and remodeling of the extracellular matrix, disrupts gap junctions and impairs cell coupling [13–15]. Moreover, the concomitant presence of obesity and metabolic risk factors may exacerbate this process via the release of proinflammatory mediators from epicardial fat [16]. Also, a recent study demonstrated that rapid atrial pacing AF was associated with changes in atrial adipocyte/adipositas-related gene expression including upregulation of 66 genes and downregulation of 53 genes at the mRNA level [17]. The clinical importance of these genes has yet to be determined. Regardless of the initiating stimulus, paroxysmal AF promotes further AF and stretch, leading to more extensive fibrosis and increased extracellular matrix deposition that ultimately slows or blocks intra-atrial conduction as a part of the adaptation to high atrial rates. In AF, the rate of disorganized atrial contraction is over 300 beats per minute or cycle length of 130–200 milliseconds while it is only 50–100 beats per minute in sinus rhythm. This high and variable rate of atrial contraction may cause significant metabolic and oxidative stress as seen in tachyarrhythmias. Interestingly, the adverse effects of AF are not limited to the atria, as a porcine model of rapid atrial pacing-induced AF produced microcirculatory flow abnormalities in the left ventricle that were associated with elevations in markers of oxidative stress but could be reversed by administration of dronedarone [18]. Ultimately, these flow abnormalities and the structural changes that initiate AF are worsened by AF itself, forming a vicious cycle that heralds progression of disease severity (i.e., “AF begets AF” [12]).

### 2.2. Electrophysiological Changes Promote Initiation and Progression of AF

In parallel with structural alterations, electrophysiological changes occur in atrial myocytes that contribute to the onset and advancement of AF. Clinical studies have shown that AF can be triggered by autonomic stimulation, bradycardia, atrial premature beats, tachycardia, accessory pathways, and acute atrial stretch. Sites of focal AF triggers include the pulmonary veins, the superior vena cava, ligament of Marshall, and coronary sinus [19]. The reason for this localization is incompletely understood but could be the result of local cardiomyocyte remodeling with altered oxidative and metabolic condition.

Electrical or focal mechanisms of AF include abnormal automaticity, triggered activity, and multiple variable reentrant circuits [1, 11]. A vulnerable atrial substrate triggers propagation of multiple reentering wavelets with heterogeneous shortening of the atrial effective refractory period (ERP) and altered conduction velocity [15]. Myocyte resting potential decreases in AF and atrial action potential morphology is altered [20]. Atrial tachycardia and stretch present in the early stages of AF alter ionic currents and promote disease progression from the paroxysmal to persistent state. Reduced L-type Ca^2+^ (*I*_CaL_) current, Ca^+^ overload, changes in K^+^ current (*I*_KACh_, *I*_K1_), Na^+^ current (*I*_Na_), and transient outward current (*I*_to_) have each been reported in AF [10]. Several pathways have been suggested to be associated with some of these changes including phosphorylation of ion channels via protein kinase A or C isoforms and effects of reactive oxygen species (ROS) [11]. However, exact molecular or subcellular mechanisms are not completely known. Increasing attention has been directed towards the role of cellular energetics and metabolism in the disease process, given the significant interaction between energetics and ionic homeostasis. Mitochondria-driven cardiomyocyte energetics and metabolism may have a significant role in regulation of ion channels [21–25]. Also, ionic currents may directly impact mitochondrial function itself, as it has been previously demonstrated that high Ca^2+^ inhibits mitochondrial respiration, dissipates membrane potential, and suppresses ATP production [26, 27]. Thus, future studies may reveal a critical role of mitochondrial-driven structural and electrophysiological changes that may have a central role in the pathophysiology of AF.

### 2.3. Cardiomyocyte Cellular and Molecular Remodeling in AF

Initial insight into the cellular structural remodeling associated with chronic AF was provided by Thiedemann and Ferrans [28], who used light microscopy to demonstrate fibrosis, myocyte hypertrophy, and myolysis in atrial tissue samples from patients with AF secondary to mitral valve disease. Several years later, these findings were supported in a canine model of mitral valve fibrosis characterized by left atrial enlargement and the common occurrence of atrial arrhythmias [29]. These animals exhibited reduced atrial wall thickness and substantial connective tissue between notably hypertrophied myocytes organized into disarranged cell bundles. More recent studies in patients with AF have extended these observations and shown sarcomere depletion and glycogen accumulation in remodeled atrial myocytes [30–32], though the concomitant presence of valve disease in the majority of subjects may have contributed to these results.

To address this limitation, experimental animal models of lone AF have proven useful to delineate patterns of structural remodeling in myocytes after prolonged periods of AF. For example, rapid atrial pacing-induced AF in dogs elicited characteristic electrophysiologic changes including shortened atrial ERP and structural alterations consisting of atrial chamber dilation and myocyte hypertrophy [33]. This approach was translated to a goat model [12], in which animals exhibited enlarged atrial myocytes and glycogen accumulation that progressively worsened with increasing disease severity [34, 35]. In addition, disorganization of sarcoplasmic reticulum, appearance of mini-mitochondria, reductions in T-tubular sarcolemmal invaginations, and dispersion of nuclear chromatin were observed [34]. Interestingly, many of the atrial cardiomyocytes reacquired characteristics of fetal cardiomyocytes, including expression of *α*-smooth muscle actin, loss of cardiotin, and a punctuated titin staining pattern [35]. It was concluded that AF was associated with myocyte dedifferentiation in the absence of degeneration, perhaps representing a conserved cellular response to stress. A more recent study [36] provided additional insight into this remodeling process in AF patients. Using a genome-wide approach to compare atrial mRNA expression in AF patients versus patients with sinus rhythm, the authors identified over 1400 genes that were deregulated in chronic AF. Functional classification analysis revealed a pattern of remodeling consistent with prominent fibrosis and metabolic adaptation to long-term metabolic stress, including upregulation of genes related to extracellular matrix composition, downregulation of contractile proteins, and coordinated transcriptional changes in metabolic enzymes favoring a shift from fatty acid oxidation to glucose utilization [36]. Additionally, atrial tissue from AF patients exhibited a general “ventricularization” characterized by substantial upregulation of ventricle-predominant genes and downregulation of atrial-specific genes, consistent with myocyte dedifferentiation and adoption of a fetal phenotype aiming at improving cell survival during extended periods of stress [36, 37].

Collectively, these results show that, aside from the atrial structural and electrical remodeling that occurs in AF, cardiomyocytes that do not succumb to apoptosis or necrosis undergo several characteristic cellular and molecular phenotypic changes. Interestingly, these alterations are reminiscent of those observed during cell dedifferentiation, including an increase in myocyte volume, myolysis, glycogen accumulation, mitochondrial changes, and chromatin redistribution [38]. Because these changes commonly occur in the absence of clear signs of degeneration, it has been suggested that atrial myocyte dedifferentiation in AF represents an adaptive, programmed cell survival response. While this idea is supported by findings in several experimental models of AF, clinical studies of AF patients that exhibit cardiomyocyte degeneration argue against this notion. These divergent observations may implicate comorbidities and/or the longer duration of disease in patients as primary factors determining the adaptive versus degenerative nature of myocyte remodeling in AF [38]. Nevertheless, our current knowledge in this area supports the notion that myocyte remodeling in AF (particularly in patients with lone AF) represents an adaptive response to stress that aims to conserve energy and promote survival, similar to what has been observed in other conditions of myocardial stress, such as the development of HM in response to chronic ischemia. Therefore, consideration of common pathophysiologic traits shared in these disease states may facilitate the development of novel therapeutic approaches by encouraging a shift in our perspective to a position that recognizes the adaptive, and potentially reversible, nature of myocyte remodeling during chronic stress.

## 3. Pathophysiology of Hibernating Myocardium

HM represents one entity along a pathophysiologic continuum describing the heart's adaptive responses to ischemia. Although complete cessation of blood flow following coronary occlusion will typically begin to elicit subendocardial necrosis as early as 20 minutes after the onset of ischemia, this type of irreversible injury is fortunately less common than reversible ischemia that produces viable dysfunctional myocardium, in the absence of necrosis, which may demonstrate transient periods of contractile dysfunction [39]. The time course of recovery is variable and may occur as early as 24 hours after return of coronary perfusion in the case of acute myocardial stunning, which is defined by reduced contractile function despite normal levels of resting perfusion [40]. If these episodes of ischemia persist, regions of viable dysfunctional myocardium may transition from chronic stunning to chronic HM, characterized by contractile dysfunction with reduced resting flow in the absence of acute ischemia or significant necrosis [41]. The clinical recognition of this tissue substrate is important because, in contrast to scarred myocardium, functional recovery of HM is possible upon revascularization or elimination of hypoxic conditions.

### 3.1. Cellular Remodeling of Hibernating Myocardium: Evidence of Myocyte Dedifferentiation

The clinical significance of HM has motivated extensive preclinical and patient-oriented research to define the cellular and molecular changes that occur in response to chronic repetitive ischemia. This investigation has revealed a pattern of remodeling encompassing structural, metabolic, and electrophysiologic changes that are similar to that previously discussed in the context of AF [39]. Structurally, hibernating myocytes exhibit myofibrillar loss, glycogen accumulation, disorganization of the sarcoplasmic reticulum, and abnormalities in mitochondrial size and shape, in concert with reactive hypertrophy and fibrosis secondary to myocyte loss [42]. Experimental large animal studies have demonstrated that this loss of myocytes results primarily from apoptotic cell death, not necrosis, with compensatory myocyte hypertrophy sufficient to preserve wall thickness [43]. Although the degree of cell loss and myocardial dysfunction stabilizes in some animal models, suggesting an adaptive response, some patient studies show an inexorable progression of cell death and functional deterioration, likely related to factors such as ongoing neurohumoral activation or the presence of comorbidities that are common in patients (as is the case with AF, discussed above). In addition to structural remodeling, HM is characterized by metabolic alterations including a reduction in oxidative metabolism and increased dependence on anaerobic glycolysis [44]. This shift contributes to a diminution in myocardial oxygen consumption that allows maintenance of energetic balance, prevents ongoing ischemia, and protects myocyte from oxidative injury. Collectively, these changes support the notion that hibernating myocytes undergo dedifferentiation towards a fetal phenotype, consistent with the observation that several embryonic/fetal gene isoforms are expressed in myocardial tissue from adults with HM, including *α*-smooth muscle actin, titin, desmin, and cardiotin [45–47].

## 4. Atrial Fibrillation and Hibernating Myocardium: Shared Pathophysiologic Traits

AF and HM share similar cellular and molecular alterations. Review of the changes observed in atrial myocytes during prolonged AF and ventricular myocytes that develop a hibernating phenotype reveals several pathophysiologic traits that are shared by the two cardiac disease states. These characteristics are summarized in Section 4.1 and collectively support the hypothesis that AF and HM each elicit the activation of a conserved adaptive response to stress. This paradigm has important clinical implications in that an improved understanding of the mechanisms driving this remodeling may facilitate the identification of therapeutic targets to promote reversion of myocytes to a mature, healthy state and restore normal structure and function. This is particularly important given the relatively recent advent of new treatment strategies for each disease, that is, revascularization of HM and restoration of sinus rhythm in AF with cardioversion. The cellular and molecular adaptations described above, and particularly their reversibility, may impact the recovery of function following these interventions, suggesting that adjunctive therapies aiming at accelerating reverse remodeling of the dedifferentiated myocyte phenotype may improve patient outcomes.

### 4.1. Pathophysiologic Traits Common to Atrial Fibrillation and Hibernating Myocardium

Consider the following:

- Apoptosis-mediated myocyte loss.
- Reactive cellular hypertrophy of remaining myocytes.
- Reexpression of fetal genes/gene isoforms (e.g., *α*-smooth muscle actin and myosin heavy chain).
- Loss and/or redistribution of structural proteins (e.g., cardiotin, titin, and desmin).
- Myolysis.
- Sarcomere depletion.
- Glycogen accumulation.
- Alterations in size and/or shape of mitochondria (smaller mitochondria).
- Downregulation of oxidative metabolism/fatty acid utilization.
- Increased reliance on glycolytic metabolism.
- Altered intracellular calcium handling.
- Redox signaling and antioxidative response.

### 4.2. Potential Mechanisms Underlying Myocyte Remodeling in Atrial Fibrillation and Hibernating Myocardium

The search for the causative factor(s) driving myocyte dedifferentiation and remodeling in AF and HM has principally focused on two potential mechanisms: myocardial ischemia and elevated wall stress. A role for ischemia is plausible in both cases; HM arises as a consequence of ischemia that typically occurs as a result of a flow-limiting coronary stenosis, while demand-induced ischemia may be apparent during AF [48]. However, the observation of myocyte dedifferentiation in remote, normally perfused regions of patients and animals with HM argues against this notion, as does the finding that a similar pattern of remodeling is apparent in nonischemic cardiomyopathy [49]. Thus, myocardial stretch may be a more likely mechanism. Indeed, coculture of adult ventricular myocytes with fibroblasts, which promotes redistribution of adhesion molecules from the distal to lateral membrane and a subsequent increase in tensile force on the myocyte, induces cell dedifferentiation despite the use of normoxic culture conditions to eliminate the possibility of ischemia [50]. Furthermore, in a pig model, cardiomyocyte dedifferentiation was observed 2 weeks after the onset of coronary stenosis but was not limited to ischemic regions, as reexpression of fetal *α*-smooth muscle actin and the loss of structural proteins were observed throughout the heart [51]. In addition, atrial stretch has been shown to promote sustained AF by prolonging the atrial ERP, suggesting that elevations in wall stress may contribute to structural and electrical changes involved in the pathophysiology of AF.

At the cellular level, alterations in calcium handling are likely an important contributor to myocyte remodeling in AF and HM. In AF, the high rate of atrial activation elicits excessive calcium influx, causing calcium overload and depression of contractile function. Impaired contractile function subsequently causes elevated preload and atrial chamber wall stress, prompting stretch-induced remodeling. In addition, calcium overload may directly promote morphologic remodeling via the activation of proteolytic pathways [38]. Support for this notion was provided by studies in a goat model of AF, which showed that elevations in calcium are transient but coincide with the timing of atrial structural remodeling [34]. Moreover, subsequent examination of heart tissue from AF patients demonstrated elevated activity of the calcium-activated proteolytic protein calpain I that correlated with ERP shortening, atrial myocyte structural remodeling, and the reduction of K^+^ channel proteins [38]. The potential mechanistic role of calcium overload is also supported by data indicating beneficial effects of calcium channel blockade during short-term AF [52–54]. However the transient nature of calcium elevations in chronic AF yields a narrow therapeutic window and the protective effect of these drugs is lost after a longer duration of AF [55].

### 4.3. Potential Therapeutic Implications

Preservation of the structural and electrical integrity of atrial myocardium is essential for the primary prevention of AF. Targeting the mechanisms that drive pathophysiologic remodeling (e.g., myocyte stretch and calcium overload) during the paroxysmal stage of AF may therefore be an effective treatment strategy if interventions can be implemented prior to myocyte loss and the concomitant deleterious structural remodeling of surviving cells that characterizes persistent AF. To decipher specific targets for treatment, it is important to develop an improved understanding of the molecular changes that contribute to the transition from paroxysmal to persistent AF. In this regard, mitochondrial dysfunction represents a promising therapeutic target given its likely role as a common pathway mediating AF and HM via metabolic and oxidative stress in association with ATP depletion, disruption of ionic currents, and increased ROS generation [56–59]. Specifically, maintenance of the functional and structural integrity of mitochondria may be efficacious to prevent AF given the important role of mitochondria in maintaining cellular energetics and ionic homeostasis [26, 27, 60]. This approach may also provide additional benefits beyond currently used catheter ablation techniques that may eliminate the source of abnormal impulse formation but do not address the myocyte remodeling that may contribute to AF generation and progression. Catheter ablation isolates pulmonary veins which are a primary source of premature atrial contraction and triggered activities. Improved understanding and treatment of myocyte remodeling in atrial myocardium and pulmonary sleeve may prevent triggers, reentry, and ectopic activities. Future studies testing interventions targeting mitochondria are warranted to determine whether attenuation of oxidative stress and preservation of mitochondrial bioenergetics may prevent or reverse atrial electroanatomic remodeling in AF, particularly at the earlier stages of disease progression.

Particularly in persistent AF, apoptosis-mediated atrial myocyte loss may play a key role in limiting the return of normal myocyte function despite the potential reversibility of cellular and molecular changes in individual surviving myocytes. From this perspective, therapies aiming at replenishing lost myocytes may have beneficial effects in allowing reverse remodeling of atrial myocytes after cardioversion, leading to sustained restoration of sinus rhythm. This paradigm is supported by a recent study examining the effect of percutaneous revascularization of HM in a well-validated large animal model of chronic ischemia. Although revascularization stimulated myocyte proliferation in previously ischemic areas of the left ventricle, reductions in contractile and metabolic proteins persisted and newly formed myocytes appeared immature, suggesting that the delayed reversal of cellular and molecular remodeling may contribute to a protracted time course of functional recovery after blood flow restoration [61]. Thus, the implementation of therapies aiming at regenerating functional myocytes via administration of exogenous stem cells or activation of endogenous myocyte progenitors may accelerate recovery of revascularized HM. In the case of AF, these novel therapeutic approaches could be coupled with restoration of sinus rhythm to promote regression of atrial myocyte hypertrophy and reversion of remodeled cells to a normal atrial myocyte phenotype, hopefully resulting in sustained maintenance of sinus rhythm and interruption of the dangerous progression of disease. Furthermore, it is possible that AF-induced myocyte dedifferentiation may actually facilitate myocyte regeneration in light of evidence that dedifferentiated adult myocytes exhibit downregulation of cell cycle inhibitors and reexpression of cardiac progenitor cell markers, collectively contributing to an enhanced ability to proliferate and form new myocytes [62, 63].

## 5. Conclusions

In summary, accumulating evidence supports the notion that AF induces a program of atrial myocyte remodeling that shares several characteristics to that observed in ventricular HM, suggesting that myocyte dedifferentiation towards a fetal phenotype represents a conserved adaptive response to chronic stress. Further understanding of the molecular mechanisms underlying these remodeling processes and recognition of their potentially reversible nature, particularly at the early stages of AF, may facilitate the development of novel therapeutic approaches to effectively prevent or treat AF and reduce the significant public health burden associated with the disease.
